# A simple method to prolong the service life of radioactive sources for external radiotherapy

**DOI:** 10.1120/jacmp.v15i4.4789

**Published:** 2014-07-08

**Authors:** Yingjie Xu, Yuan Tian, Jianrong Dai

**Affiliations:** ^1^ Department of Radiation Oncology Cancer Institute & Hospital, Chinese Academy of Medical Sciences Beijing China

**Keywords:** radioactive source, service life

## Abstract

A radioactive source is usually replaced and disposed after being used for a certain amount of time (usually a half‐life). In this study, a simple method is proposed to prolong its service life. Instead of replacing the used source with a new source of full activity, a new source of less activity is added in the source holder in front of the used one, so that the total activity of two sources is equal to the initial activity of the used source or even higher. Similarly, more sources can be added to the previous ones. Attenuation of front source(s) to the back source(s) was evaluated with exponential attenuation equation, and variation of source‐focus distance (SFD) with inverse square law for Leksell 4C Gamma Knife, which served as an example of external radiotherapy units. When the number of front sources increased from 1 to 3, the relative air kerma decreased from 36.5% to 5.0%. Both the attenuation effect and SFD variation contributed to the decrease in air kerma, with the former being the major factor. If the height of the source can be decreased in some way, such as increasing the specific activity of sources, the sources can be used more efficiently. The method prolongs the service life of sources by several factors, and reduces the expense of source exchange and reclamation.

PACS number: 87.56.bg

## I. INTRODUCTION

Since the middle of the twentieth century, radioactive sources (mainly cobalt‐60) have been widely used in teletherapy, like the cobalt teletherapy machine and the Gamma Knife. Compared with linear accelerators, cobalt teletherapy units have some merits such as less consumption of electric power, more stable beam, easier to manage, and minimum infrastructure requirements. The major problem with such units is the periodical replacement and disposal of decaying sources. In developed countries, the cobalt teletherapy machines are no longer recommended, compared to linear accelerators, for modern radiotherapy. But because of its reliability and simplicity in maintenance, cobalt teletherapy units are still in widespread use worldwide, especially in developing countries like China and India.^1^, ^2^ A survey^(3)^ in 2011 about the radiotherapy equipment used in mainland China showed that 286 cobalt units are still in use. Fox et al.^(4)^ compared the cobalt teletherapy machine and linear accelerator, and demonstrated that the commercial cobalt source with a divergent MLC for IMRT can achieve nearly identical treatment plans, compared to 6 MV X‐ray IMRT. They also concluded that the cobalt penumbra is not inferior to linac penumbra for MLC‐based IMRT.

Gamma Knife units also use cobalt sources. They concentrate the radiation coming from dozens of sources, even hundreds of sources, and give a very high dose to the focus. They are widely used in treatment of intracranial lesions and some metastases in lung or liver.

Because of source decay, patient treatment times will be continuously prolonged for the same treatment doses starting from the time that new sources are installed. To avoid treatment times that are too long, the sources have to be replaced after being used for some period of time, usually a half‐life. However, the sources still have half of their initial activity, and this half activity is disposed by a professional company, thereby incurring a financial expense. In recent years, governments have placed stricter regulations on radioactive sources because of concerns about terrorism; thus, the sources are becoming more and more expensive, and so does the disposal of consumed sources.

There has been little effort on making more use of the sources over the last several decades,^(5)^ although studies by the Schreiner group have looked at improved source design for cobalt‐60 tomotherapy applications.^(6)^ In this study, we propose a simple method to extend the service life of radioactive sources, and we evaluate its performance with an exponential attenuation equation and the inverse square law.^(7)^

## II. MATERIALS AND METHODS

### A. Method description

To prolong the service life of sources in cobalt units, a new design of source holder in the treatment head is presented. The source holder is a few times the source height such that it is capable of holding a few sources rather than just one. It can be used in single source systems like cobalt teletherapy units, and in multisource systems such as the Gamma Knife (Elekta, Stockholm, Sweden). After a half‐life, the source is not removed; instead, it is just moved backwards, or put into another holder (when it is a multisource system), and a new source is added in the original position. In this way, the source that used to be removed can still be kept in use in the treatment unit.

**Figure 1:**
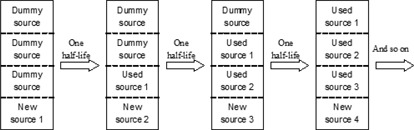
Schematic diagram of how the source is moved after one half‐life.

Figure 1 is a source holder of a single source system. It is assumed that there are four source positions in a holder. At the very beginning, only the first source (New source 1) is in the holder with three dummy sources used as fillers. After one half‐life, a dummy source is removed, and the other sources are moved backwards by one source position. The new source (New source 2) should be placed in the vacancy left by source 1. After the second half‐life, the process would be repeated. Similar steps are performed for sources 3 and 4.

**Figure 2:**
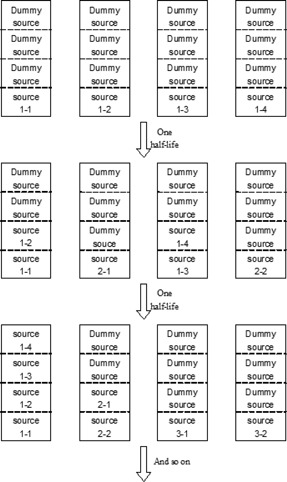
Schematic diagram of how to combine the sources. The sources are identified with two numbers. The number before hyphen represents the batch number; the number following hyphen is the successive number of the source in the same source batch (e.g. source 1‐2 is the second source from the first source batch).

Figure 2 is a schematic diagram for the multisource system, assuming four sources are contained in one group. At first, the real sources will be placed in the positions closest to the exit window of the source holders. Three dummy sources will be used as fillers for fixation (see the first row of Fig. 2). After one half‐life, four sources will be combined in two groups, and two new sources will be set to the vacancies. After the second half‐life, the four sources assembled in first batch will be combined together into one holder, and the two source put in second batch will be set to another holder. Another two new sources will be placed in the vacancies. After the third half‐life, the four sources placed in first batch can be removed, and the process can be repeated.

### B. Performance evaluation

#### B.1 Effect of source attenuation

According to the new method, when the activity of a source is reduced, the source will be moved backwards and a new source will be set in the vacancy. Contribution of the old source will be reduced because of the attenuation of the new source. Considering that the distance from the source to the focus is much larger than the source dimension, the linear attenuation equation^(7)^ was used to evaluate the attenuation. The function is:

(1) $$S_{k}^{'}(t)=S_{k}(t)\times e^{-\mu_{source}\times nh_{source}}\times e^{-\mu_{shell}\times nh_{shell}}$$

where *t* is the decay time of the source, $S_{k}$ is the air‐kerma strength of the source when it is positioned just behind the exit window of source holder, $S_{k}$’ is the air‐kerma strength of the same source when it is positioned posteriorly, $\mu_{source}$ is source attenuation coefficient, $\mu_{shell}$ is the attenuation coefficient of the source shell, $h_{source}$ is the source height of the radioactive material, *h*_*shell*_ is the height of the source shell including in front of and behind the radioactive material, and *n* is the number of sources.

The source size of Leksell 4C Gamma Knife^(8)^ was chosen here as an example for evaluation. *μ*_*sourc*_ */ρ*_*source*_ is mass attenuation coefficient of 1.25 MeV gamma rays and equal to 0.0527 cm^2^/g. For cobalt sources, pellets are packed with a fair amount of air in between, so here $\rho_{source}$ is the effective density, which is 5.88g/cm^3^.^(6)^ $h_{source}$ is the height of the active source and equal to 2 cm. The outer shell is stainless steel ($\rho_{shell}=7.85g/{\text{cm}}^{3}$), the value of $\mu_{shell}/\rho_{shell}$ is 0.0535 cm^2^/g, and the value of $h_{shell}$ is 0.61 cm (the front layer of radioactive material is 0.12 cm, the back layer of radioactive material is 0.49 cm).

#### B.2 Effect of SFD variation

Samat et al.^(9)^ compared the exposure rate, X, calculated by the point source formula and a formula used for a finite size source. The point source formula uses the inverse square law. They found that a negligible deviation (0.1%) between these two formulae if $d/h_{source}\geq 5$, where *d* is distance measured from the center of the source to the measured point and $h_{source}$ is the height of the cylindrical source (cm).

In this new design of the source holder, the spent source will be moved backwards, and the distance between the source and the focus become larger. Using the size of Leksell 4C Gamma Knife^(8)^ as an example, d=40.1 cm, $h_{source}=2\;\text{cm}$, and $d/h_{source}\geq 5$, where *d* is the distance between source and focus, and $h_{source}$ is the height of the radioactive source. The inverse square law is used here to evaluate the effect of SFD variation,

(2) $$S_{k}^{''}(t)=S_{k}^{'}(t)\times [d^{2}h(d+n{l)}^{2}]$$

Here $S_{k}$ *”* is the air‐kerma strength of the source after considering the effect of SFD variation, *d* is the distance between source and focus, and *h* is the height of the source, which equals 2.72 cm.

### C. Monte Carlo simulation

**Figure 3:**
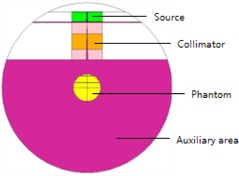
Illustration of geometry definition of the whole simulation model which included four components: source, collimator, phantom, and auxiliary area.

To validate the equations described above, Monte Carlo N‐Particle Transport Code (MCNP) ^(10)^ was also performed for new designs of source holder. A simple model was built according to the single source channel of the Elekta Leksell 4C Gamma Knife, which included four components: source, collimator, phantom, and auxiliary area (Fig. 3). The basic data for source and collimator were adopted from the paper by Al‐Dweri et al.^(8)^ To get authentic data, $2\times 10^{9}$ particles were sampled during each simulation. In order to shorten the computation time, parallel computation of MCNPX (Monte Carlo N‐Particle eXtended) based on Message Passing Interface (MPI) environment was employed.

## III. RESULTS & DISCUSSION

Using the air‐kerma strength of a source when it seated at the exit window of source holder as reference, the remaining relative air‐kerma strengths of this source compared to itself were calculated when there were 1, 2, and 3 sources in front of it (Table 1). The results of Monte Carlo simulation were also listed in Table 1. The difference between the two kinds of results was no more than 2.4%.

It can be seen that the remaining relative air‐kerma strengths decreased quickly with an increase in the number of sources in front of the specified source. When the number of front sources increased from 1 to 3, the relative air‐kerma strengths decreased from 34.4% to 4.3% in Monte Carlo simulation. Both the attenuation effect and SFD variation effect contributed to the decreasing of air kerma, with the former being the major factor.

Both effects depend on the source height. On condition that the total source activity remains for a treatment unit, there are at least four possible methods to decrease the source height. The simplest method is to decrease the height and increase the diameter of the source. This has the disadvantage of enlarging the beam penumbra. The second method is to increase the packing factor of cobalt‐60 pellets, as suggested by Joshi et al.^(6)^ and George et al.^(11)^ The third method is to increase the number of sources. For example, a typical cobalt teletherapy unit only has one source, whereas an old unit designed for parallel‐opposed pair treatments by Cunningham et al.^(12)^ has two sources in two heads, and a newly designed cobalt‐60 teletherapy system, the ViewRay system (http://www.viewray.com/favicon.ico) has three sources in three heads. This approach has a disadvantage in that the treatment unit head has to be redesigned. The fourth method is to produce purer sources so as to increase the specific activity of sources. Today the specific activity of the sources in commercial use is about 250–300 Ci/g. Boswell reported that a Co‐60 specific activity of 700 Ci/g was achieved in production of Savannah River reactors. ^(13)^ Even the latter is still far less than the specific activity of cobalt‐60 sources theoretically achievable, which is 1127 Ci/g.^(14)^ There are big differences among those values. That means the fourth method has the biggest potential. If the manufacturing process of cobalt sources is improved and the specific activity is higher, the sources can be used more efficiently without the above disadvantages.

It needs to be noted that the results of Table 1 are based on the attenuation of primary photons only. The impact of the increase in the number of scattered photons has not been addressed, the results of which would likely yield increased air‐kerma rates and are probably of greater significance for wider diameter sources. A quantitative evaluation of this impact will be performed in future research using Monte Carlo methods.

The idea of back‐to‐back sources presented in this paper is a concept of principle. The details would have to be worked out by the designers of teletherapy machine heads, since clearly the implementation of sources placed back‐to‐back would require some redesign of such teletherapy machines. However, it is expected that the use of old radioactive sources would provide some benefits compared to the full replacement of existing sources every five years. Still using the source size of Leksell 4C Gamma Knife as an example, using the results in Table 1 obtained by Monte Carlo simulation, after one half‐life, if each used source is moved backwards according to the schematic diagram shown in Fig. 1, the used source can give 17.2% (i.e., 50%×34.4%) contribution to the focus point; so a new source of 81.7% activity needs to be added, rather than a source of full activity. That means 17.2% saving on activity cost and no source is withdrawn. On the other hand, if the used sources are combined according to Fig. 2, after one half‐life time, every two used sources are combined to give 67.2% activity (i.e., 50%+50%×34.4%) of the original source strength, and two new sources need to be added. To maintain the same dose rate at the focus point, the activity of each new source is 132.8% of the original source activity. Therefore, the saving on source activity is 33.6% (i.e., (200%‐132.8%)/2). Besides that, since the number of new sources is half of the total number of source holder, there is 50% saving on source encapsulation cost.

**Table 1:** The remaining relative air‐kerma strength of a source compared to itself with sources in front of it

*The Number of Front Sources*	*1*	*2*	*3*
Attenuation correction	0.416	0.173	0.072
Distance variation correction	0.877	0.775	0.690
Total correction (Equation)	0.365	0.134	0.050
Total correction (Monte Carlo simulation)	0.344	0.122	0.043

In spite of above benefits, implementing the proposed method does have costs. First, the treatment head needs redesign, and its size and weight increased. That may affect the isocenter precision, if the treatment system is a single‐source C‐arm teletherapy machine, or the focus precision, if the system is multisource stereotactic therapy system. Second, beam parameters of combined sources (used sources, or used source(s) with a new source) is somehow different from that of single new source. That means more work on measurements and beam modeling. Furthermore for multisource system, not only the beam parameters and modeling of multiple combined sources are more complex, combining sources would require source vendors to customize source activity based on the existing source activity and source positioning (if applicable). The dose rate would vary, depending on how the used and new sources are arranged. Third, compared to replacing a used source with a new source, it takes more time to add a new source in front of a used source or combine two used sources. Therefore, further cost‐benefit analysis is necessary.

## IV. CONCLUSIONS

A simple method is proposed to prolong the service life of radioactive sources. Through designing a few source positions rather than one position in the source holder, old sources can be moved backwards rather than removed when a new source is added. The method prolongs the service life of sources by a few times, and reduces the expenses of source exchange and reclamation. Four possible methods to improve the estimated performance are discussed.

## ACKNOWLEDGMENTS

This work was supported in part by National Natural Science Foundation of China (Grant No. 11275270) and National Key Technology R&D Program (2011BAI12B05). The authors are thankful to Dr. Jacob (Jake) Van Dyk, Professor, The University of Western Ontario, for inspiring comments on this paper.
